# Germ-free, carefree: injured skin uses IL-24 to kick-start repair independent of pathogen-recognition

**DOI:** 10.1038/s41392-023-01609-y

**Published:** 2023-10-04

**Authors:** Ruben A. Ferrer, Marta Torregrossa, Sandra Franz

**Affiliations:** Department of Dermatology, Allergology and Venerology, University Leipzig, Leipzig, Germany

**Keywords:** Cell biology, Stem cells

A recent paper published in *Cell*, Lui et al. shows that epidermal stem cells respond to injury-induced hypoxia via upregulation of IL-24 to coordinate repair in a sterile fashion by autocrine and paracrine signaling on keratinocytes, fibroblasts, and endothelial cells.^1^ The finding of this study is of major significance as it identifies for the first time a signaling pathway that translates injury into tissue repair independently of pathogen-sensing.

**Fig. 1:**
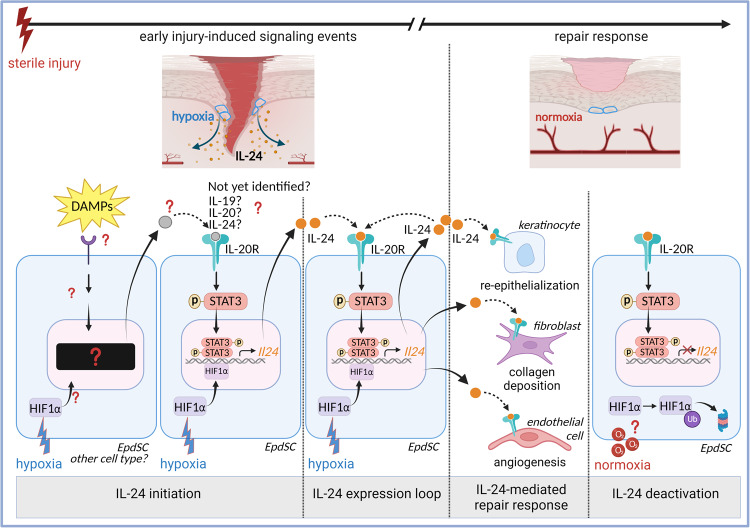
Injury-induced IL-24 signalling in epidermal stem cells elicits tissue repair in skin wounds in mice independently of pathogen-sensing and immune activation. IL-24 initiation: Tissue injury induces hypoxia and other damage-associated molecular patterns (DAMPs) that are produced from the injured tissue to indicate its “non-homeostatic” state. Epidermal stem cells (EpdSCs) and potentially other cell types at wound margins sense these injury-induced signals early upon wounding and release signals to EpdsSCs to initiate IL-24 activity. Upregulation of *Il-24* in EpdSCs requires two signals: (i) hypoxia-induced HIF1α-stabilization; (ii) IL-20 receptor (IL-20R) signaling and STAT3 activation. The first signal engaging IL-20R remains elusive, but may involve IL-24, IL-20, IL-19 or unknown factors produced by EpdSCs or other cells in response to injury. IL-24 expression loop: HIF1α-stabilization and IL-24/IL-20R/STAT3 activation converge in EpdSCs in the migrating epithelial tips at the wound edge to feed an autocrine signalling loop. This causes further upregulation of *Il-24* required to initiate repair. IL-24 mediated repair response: IL-24 emanating from EpdSCs targets IL-20R expressing tissue cells to coordinate a repair response. This includes the induction of keratinocyte proliferation to allow re-epithelialization, the activation of fibroblasts for dermal repair with collagen deposition, and the stimulation of endothelial cells for angiogenesis. IL-24 deactivation: Restoration of physiological oxygen levels (normoxia) with destabilization of HIF1a and potentially other unknown signals appearing once repair has been initiated should deactivate *Il-24* expression in EpdSCs. Underlying mechanisms remain to be identified. *Graphic was created with BioRender.com*

Skin injury releases damage-associated molecular patterns (DAMPs) represented by tissue-components and intracellular contents as well as changes in the levels of certain molecules and metabolites that signal the “non-homeostatic” state of the tissue. In addition, microorganisms entering a wound release molecules, which are sensed as “non-self” (pathogen-associated molecular patterns, PAMPs). Together, DAMPs and PAMPs initiate a complex and coordinated repair process aimed at restoring homeostasis. The exciting work from Liu et al. reports of an additional novel mechanism by which a “non-homeostatic” state in murine cutaneous wounds elicits a response through IL-24 to mediate repair (Fig. 1).^1,2^

Liu et al. perform gene expression analysis from skin cell populations as well as in situ studies to identify STAT3 activation in epidermal stem cells (EpdSCs) in the migrating epithelial tip within wounds.^1^ There is a strong induction of IL-24, followed by IL-19, although to lesser extent, both of which can activate STAT3.^1^ IL-24 is a conserved member of the IL-10 family, which also include IL-19 and IL20; all of them signal through IL-20-receptors (IL-20R).^3^ The main source of IL-24 in the wounds are the EpdSCs.^1^ The authors provide evidence for IL-24 upregulation occurring with absent PAMP-recognition or Toll-like receptor (TLR) signaling.^1^ Previous studies in injury models show nevertheless, that various cell types can upregulate IL-24 and this occurs also as response to inflammatory cytokines engaging STAT1 and TLR-signaling.^3^ STAT1 is downstream of interferons, which in turn can be upregulated after sensing of DAMPs and PAMPs. This raises the question whether interferons induced by DAMP-sensing and independently of PAMPs/TLRs also start IL-24 signaling. From the evolutionary perspective, IL-24 and its receptors share close sequence and structure homology with interferons. This interesting finding by Lui et al. suggests that IL-24 may go beyond a mere “non-homeostatic” sensor and act at the interface of inflammation and repair programs.

Expression of IL-24 requires hypoxia-induced stabilization of HIF1α and autocrine activation of IL-20R and STAT3 by IL-24.^1^ What and which cells provide the first signal to initiate this positive feedback loop remain unknown. Possible candidates involve other factors that signal through IL-20R such as IL-19 and IL-20, with IL-19 being also upregulated in EpdSCs.^1^ It would be interesting to explore the induction of these candidates in the first hours following injury. Of note, hypoxia-signaling is not the only way in which skin and other tissues sense a disturbance in homeostasis. Sensing of other DAMPs could also have initially activated IL-24 in the system.

Il-24 is required to mediate repair by autocrine and paracrine effects on other cell types within wounds. Mice with constitutional *Il24*-deletion (*Il24*^*−/−*^) present a delayed wound healing course with impaired STAT3 activation in EpdSCs at the wound edge and reduced thickness of the epidermal layer.^1^ Mice lacking IL-20RB (*Il20rb*^*−/−*^) show an even more marked defect, probably due to lack of signaling from both IL-24 and IL-19.^1^ Impact of *IL19*-deletion alone was nevertheless not assessed. Both *Il24*^*−/−*^ and *Il20rb*^*−/−*^ mice showed marked paucity of dermal blood vessel angiogenesis.^1^ *Il24*^*−/−*^ mice also showed reduced fibroblast-numbers and collagen deposition as well as defective macrophage differentiation and mobilization.^1^ These actions of IL-24 in repair seem to be partially mediated by IL-20R binding of IL-24 and hypoxia sensing in EpdSCs through the HIF1α-target *Slc2a1*. This gene encodes glucose transporter protein type 1 (GLUT1) and is expressed in EpdSCs at the migrating tip in wounds.^1^ Mice with defective IL-24/STAT3 signaling (*Il20rb*^*−/−*^, *Il24*^*−/−*^, *Krt14/STAT3*^*−/−*^) show reduced GLUT1-expression in wound-epithelium and inducible *Glut1*-deletion in wound-EpdSCs results in defective dermal angiogenesis and fibroblast activation.^1^ GLUT1-expression also changes with alterations in metabolism, glucose levels and energy-rquirements within a tissue. It might be that GLUT1 upregulation in wound-EpdSCs is mediated by IL-24/hypoxia as well as metabolic changes caused by injury. The observations from Liu et al. contribute to the notion that metabolic changes are an important “loss of homeostasis” signal, which participate in the response to tissue-injury.^4^

Importantly, the authors make the striking observation of an initiation of a repair response in unwounded mouse skin by ectopic expression of Il24.^1^ Here, tissue changes were unleashed in complete absence of DAMPs and PAMPs, reinforcing the notion of an important role for IL-24 signaling for kick-starting events of repair. This suggests that IL-24 is required for fibroblast proliferation and collagen production, proliferation of epithelial and endothelial progenitors independent of damage and pathogen-sensing. Whether this is the case in humans, it remains to be studied. IL-24 and its receptors are expressed and induced in human wounds, but there are conflicting reports of an inhibitory effect on keratinocyte migration and proliferation.^5^ Also contradictory are reports on the effect of IL-24 on vessels, with most data showing an induction of proliferation but blocking of differentiation.^5^ IL-24-induction has been implicated in the pathogenesis of psoriasis vulgaris, airway damage in severe COVID-19 and in ulcerative colitis, raising the question whether IL-24 serves as an eminent signal for repair in organs with epithelial layers. Psoriasis shows hyperproliferation of keratinocytes and capillaries as well as defective differentiation. Another cutaneous condition, Pyoderma gangrenosum, presents with non-healing ulcers after none/minimal-trauma with excessive epidermal and vascular proliferation and defective tissue-maturation and resolution. Non-healing leg ulcers also show hypertrophy/hyperplasia of the epidermis and increased number of vessels in a hypoxic wound environment. It is worthy to explore whether defective IL-24 is implicated in the pathophysiology of these diseases. Could excessive IL-24 cause constant proliferation and maintain the tissue in a “constant repair” state hindering maturation and resolution? Anecdotally, hyperbaric oxygen has been used to treat non-healing leg ulcers. Could it be that by removing hypoxia, IL-24 goes back to pre-injury levels and this allows resolution? This also raises an additional question: What deactivates IL-24 in normal wounds? Restoration of normoxia? Clearance of DAMPs/PAMPs? On the other side of the coin, it could also be questioned, whether human diseases with repair-impairment are caused by deficient IL-24 activity. In this regard, IL-24 may represent a promising therapeutic target, either by supplementing IL-24 (when low levels are responsible for disease) or by blocking IL-24, perhaps simply by restoring normoxia (when excessive levels cause or perpetuate disease). Since the epidermis is the hub for IL-24 signaling, agents targeting IL-24 would only need to reach the epithelium and could be applied via topical treatments.
